# The Zebrafish model in dermatology: an update for clinicians

**DOI:** 10.1007/s12672-022-00511-3

**Published:** 2022-06-17

**Authors:** Irene Russo, Emma Sartor, Laura Fagotto, Anna Colombo, Natascia Tiso, Mauro Alaibac

**Affiliations:** 1 Unit of Dermatology, University of Padua, Via Gallucci 4, 35128 Padua, Italy; 2 Department of Biology, University of Padua, Via U. Bassi 58/B, 35131 Padua, Italy

## Abstract

Recently, the zebrafish has been established as one of the most important model organisms for medical research. Several studies have proved that there is a high level of similarity between human and zebrafish genomes, which encourages the use of zebrafish as a model for understanding human genetic disorders, including cancer. Interestingly, zebrafish skin shows several similarities to human skin, suggesting that this model organism is particularly suitable for the study of neoplastic and inflammatory skin disorders. This paper appraises the specific characteristics of zebrafish skin and describes the major applications of the zebrafish model in dermatological research.

## Introduction

Since their use, animal model systems have offered a technical means to perform studies that could not be otherwise undertaken in human subjects. They represent a fundamental part of research and offer precious keys to understanding human physiology and pathophysiology. Moreover, not only do they allow for more advanced pharmacokinetic and pharmacodynamic studies, but also for the discovery of new treatments for human diseases.

Because of their well-known physiological and genetic similarities to humans, murine and other mammalian models have been routinely used for medical research. However, other animal models are showing distinct advantages over these conventional models. Therefore, interest in this field has increased over recent years.

Among the animal models studied thus far, fish seem to be the most interesting non-mammalian vertebrate model, because of their low maintenance costs and *ex uterus* development of progeny that allows for in vivo imaging. For instance, Platyfish (*Xiphophorus*) and Medaka (*Oryzias latipes*) have already been successfully used as models to study melanoma [1–3].

## The zebrafish organism

The zebrafish (*Danio rerio*) was first introduced as a model for genetic studies by Streisinger and colleagues in the early 1980s [4]. Zebrafish are small vertebrate tropical fish characterized by low-cost maintenance and exploited as a model for both reverse and forward genetic studies.

At first, zebrafish were used as a model in forward genetic studies (the identification of a specific genotype through observation of a certain phenotype). This was the case in N-ethyl-N-nitrosurea (ENU) induced mutagenesis used to produce point mutations, followed by extensive phenotypic screening. However, forward genetic screens using this method were time consuming and laborious [5, 6].

Fortunately, increasingly advanced techniques in recent years have allowed to overcome the challenges of creating several disease models using zebrafish and to expand zebrafish studies through the reverse genetics (the observation of the phenotype produced by a known genotype). Moreover, studies have proved that there is a high level of similarity between human and zebrafish genomes, estimating that 70% of human genes have at least one zebrafish ortholog. These data are astonishing, especially if we consider that a mouse shares about 80% of its genome with humans [7, 8]. Moreover, over 80% of known human disease genes, including oncogenes and tumor suppressor genes, have their orthologues in zebrafish and several pathways are also conserved, even those implicated in carcinogenesis [8].

All these characteristics and evidence have boosted the use of zebrafish as a model for understanding human genetic disorders, including cancer, and have pointed out their potential for in vivo screening for new therapies, which is especially important in our era of personalized medicine.

Therefore, although mouse models remain the most used in the medical research field, the zebrafish has several advantages and unique features that murine models do not have, which explain its ancillary and complementary role.

The advantages of using zebrafish as a model are numerous, going beyond their low-cost maintenance and small size. They display a high fecundity, with the ability to fertilize about 200–300 eggs every 5–7 days. Fast ex utero development together with the embryos’ optical clarity allow for the observation of early physiological and/or pathological development by in vivo direct cell imaging [9, 10]. In addition, the *casper* zebrafish was introduced as a genetic strain intentionally created to maintain transparency throughout adult life, making it even more affordable to study cancer cells’ behavior in a living organism [11].

Cell-based assays for the study of the absorption, distribution, metabolism and toxicity of compounds and drugs give only limited information, whereas pharmacologic molecule screening in zebrafish might help to overcome this problem. Parallel physiological responses have been observed in the use of drugs and small molecules in zebrafish and mammal models [9]. Drug screenings benefit from both the embryos’ transparency, which makes it easy to collect imaging data after treatment, and the high throughput assays, which are made possible by the female’s ability to lay many eggs (about 10,000 eggs per annum). This means that imaging, cellular analysis and advanced statistics can be performed simultaneously in an incredibly large number of fish, with laboratory space being the only limiting factor [12, 13]. These premises explain the zebrafish’s potential role as a bridge between cell-based assays and biological validation of a certain compound.

Moreover, zebrafish might represent a clue in the attempt to identify therapeutic targets for the treatment of human diseases, which remains a big challenge in medical science [14]. In phenotype-guided drug studies, the presence of phenotype alterations in the whole organism may suggest the effectiveness of a drug, even when the target is unknown. This approach helps both the development of new drugs and the simultaneous identification of the molecular pathways underlying the disease that are inducing that specific phenotype [15]. Compared to mice, zebrafish studies enable us to analyze a greater number of phenotypes at reduced costs and labor.

Even though this fish model is extremely versatile in medical and especially in pharmacological research, there are a few drawbacks that should also be pointed out. Firstly, the zebrafish is a poikilothermic fish that needs to be bred in an environment with a temperature around 28 °C to survive. This differs from mammals’ homeostatic temperature, thus hindering studies where temperature is a determining factor. However, it can tolerate a wide range of temperature variation, spanning between 6 and 38 °C, for limited periods of time [16]. Secondly, teleost genome duplication involves the presence of genes in more than one copy (paralogs), which might hamper molecular genetic studies. Lastly, another disadvantage of the zebrafish is the scarcity of available antibodies that specifically target zebrafish proteins, and the technical difficulty in raising antibodies against zebrafish targets [17–19]. This is especially relevant for cell surface and secreted proteins, since immunogenic glycans on zebrafish extracellular proteins hamper elicitation of protein-specific antibodies in mammals used for raising such antibodies [20].

### Zebrafish skin

Fish skin comprises the epidermis, dermis, and hypodermis, thus resembling mammalian skin. However, unlike mammals’ and terrestrial vertebrates’ epidermis, which is covered by an outer layer of keratinized dead cells, zebrafish skin surface is made of living cells that are covered with mucus and lacks a cornified envelope [21]. Furthermore, zebrafish has no mammalian appendages, since hair follicles and sebaceous glands cannot be detected. However, zebrafish presents the breeding tubercle, which is an epidermal appendage shared with mammals [22].

Mammalian epidermis is a well-organized stratified tissue that includes basal, spinous, granular, and horny cells from the basal membrane to the skin surface. Teleost epidermis only has three layers [23]. The surface layer is a single cell layer in which cells are rich in keratin filaments and are continuously replaced at their death, without producing a stratum corneum. The intermediate layer is composed of different cell types, including unicellular glands (mucous cells and club cells), sensory cells, ionocytes and undifferentiated cells. The basal layer is a single cell layer which is attached to the basement membrane via hemidesmosomes, which tightly link the epidermis to the dermis [24].

Maturation of zebrafish from embryo to fully developed fish only takes a few days. Layers representing the epidermis and the dermis are already detectable at one day post-fertilization (dpf). In adult zebrafish, scales covering the epidermis form at around the 30th dpf and sonic hedgehog pathway has been identified has having a role in their development [25]. Collagenous stroma formation is dependent on fibroblasts, whereas pigment production derives from melanocytes, belonging to neural crest-derived pigment cell system [26].

Several epidermal marker genes, including keratins 1 and 5, the 230 kDa bullous pemphigoid antigen, plectin, and several cutaneous basement membrane zone (BMZ) genes, including type IV, VII and XVII collagen, are expressed in zebrafish skin in early developmental stages. Most expressed human collagens types, including collagens I, V, and VI, are detectable in zebrafish skin from 6th dpf. In conclusion, zebrafish repertoire of genes involved in cutaneous development reveals strong similarities with human skin [25].

**Fig. 1:**
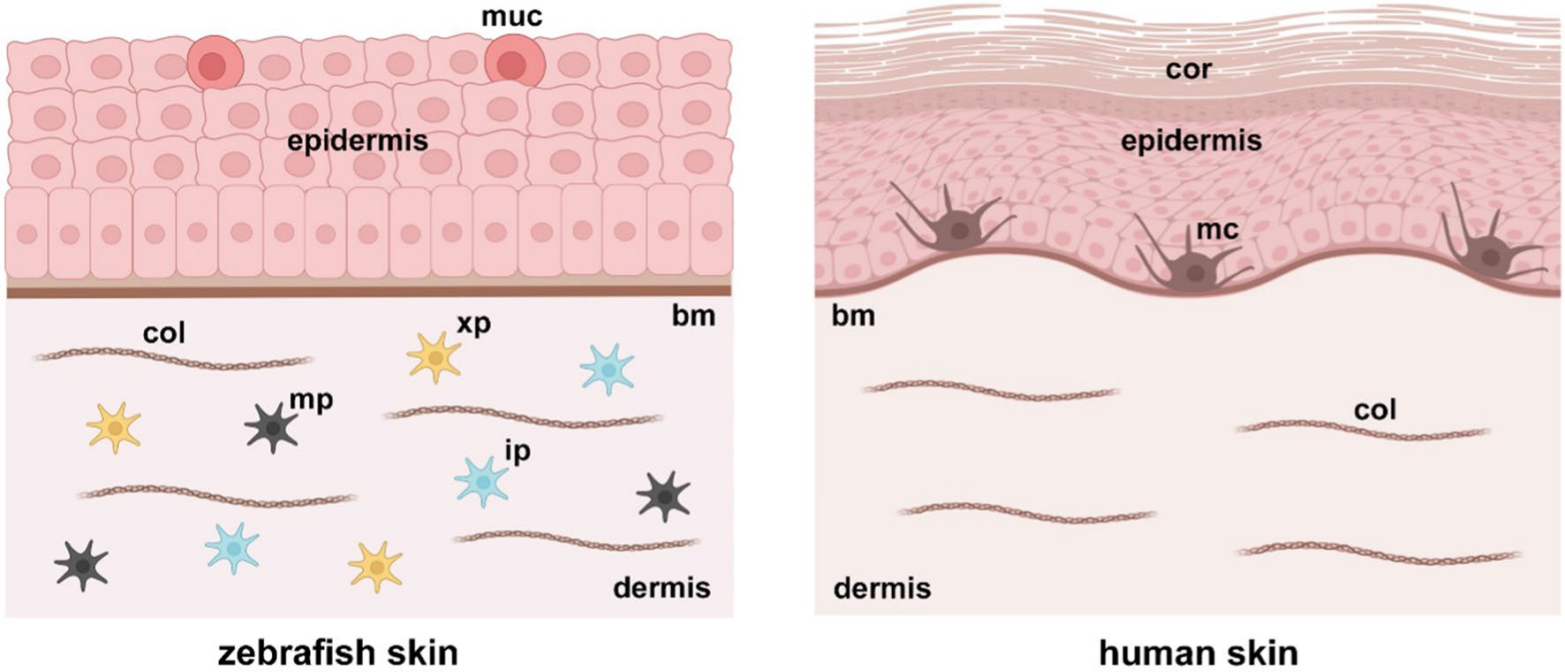
Comparison between zebrafish and human skin. Both zebrafish and human adult skin include a multi-layered epidermis, a basal membrane (bm) and an underling dermis containing collagen (col) fibers. Zebrafish epidermis contains mucous cells (muc), while human epidermis has a stratum corneum (cor) as outermost layer. Zebrafish pigment cells include xanthophores (xp), iridophores (ip) and melanophores (mp); human pigment cells are represented by melanocytes (mc). Images created with BioRender.com

The zebrafish neural crest produces three different kinds of pigment cells: melanophores, xanthophores and iridophores (Fig. 1). Melanophores synthesize melanin and are analogous to melanocytes of vertebrates, xanthophores have a yellow appearance caused by pteridine pigments, and iridophores contain iridescent platelets which reflect light. Melanophores firstly develop among pigmented cells at approximately 24 h post fertilization from melanogenic progenitors deriving from the neural crest [27].

Like in mammalian melanocytes, the tyrosine-protein kinase KIT has a major role in promoting the initial migration of melanocytes in the first two days of the embryos [28]. Later in zebrafish larval development, a new set of melanocytes contribute to formation of stripes characterizing adult zebrafish after metamorphosis [29].

### Skin inflammation in zebrafish

Skin is essential in defending fish from environmental stress factors. Since fish are poikilotherms, even small changes in the external parameters may lead to injury and inflammation [30]. A series of epithelial cells, resident non-immune cells, vascular endothelial cells and mucosal epithelial cells help initiate and coordinate the inflammatory response [31–33]. Interestingly, unlike humans, [34]. fish do not have major lymphoid accumulations. It is still unclear where fish lymphoid cells naturally reside. The most probable theory is that fish leukocytes migrate to the skin via mucus secretions in response to damage stimuli [35–37].

Inflammation pathways are regulated by the NF-κB family transcription factor both in mammals and fish [38] and classic pro-inflammatory cytokines such as IL-1β, TNF-α and IL-6 prevail as paralogues in most teleosts [39–41], thus making the main mechanisms of inflammation similar in the two species. Neutrophils play an essential role in initiating the inflammatory response in both mammals and zebrafish and in perpetration of the inflammation, especially via TNF-α and IL-1β [42–48].

Once activated, monocytes differentiate into classically pro-inflammatory macrophages, functioning as antigen presentation cells and producing reactive oxygen species (ROS), TNF-α and IL-1β [42, 49–52].

Besides, neutrophils activation determines exocytosis of their granules [42, 50, 51, 53, 54]. Antigen presenting cells such as dendritic cells, macrophages and endothelial cells are also recruited by neutrophils [51]. While in mammals leukocytes originate in the bone marrow and mature in lymph nodes, zebrafish lacks such structures [55]. Specifically, the bone marrow has its counterpart in the head kidney acting as a major hematopoietic and lymphoid organ. Indeed, the thymus, spleen and mucosa-associated lymphoid tissues (MALT) are shared between fish and mammals [56]. Migration and proliferation of the immune cells in zebrafish skin has been recently studied using fluorescent light which induced the early expression of skin genes associated with inflammation [57].

## Zebrafish as a model system in oncology

One of the biggest obstacles in the field of oncology is to address cancer heterogeneity either in inter-individual differences or in intra-tumoral contexts [58, 59]. Innovations in cancer research have largely benefited from further exploring these processes in live animals, with a strive to identify and target the most frequent driver mutations as a rational approach to treatment [60]. This is especially done in early tumor development at a cellular level.

As an example, patient-derived cancer cell xenotransplantation (PDX) could help to overcome treatment resistance due to added mutations in tumor cells, by means of large-scale drug (small molecules) screening. This process is not as easily achievable in murine models as it may be in non-mammalian models such as fish, since recipient immunosuppression is required for PDX in mice [61–63].

The zebrafish has recently caught attention due to the aforementioned characteristics, alongside its highly-conserved cancer signaling pathways compared to the human species.

Moreover, the zebrafish is a versatile model, as it is possible to operate a mutation in a specific gene, thus creating a stable transgene, or to create a transient over-expression or down-regulation of a specific gene. Forward and reverse genetic screens are also possible in zebrafish [64].

Initially, zebrafish have been used in forward genetic screens to test the effects of mutagens on neoplasm development. Ethylnitrosurea screens leading to mutations in tp53, one of most studied genes among those involved in cancer pathogenesis, were among the first experiments in zebrafish in the field of oncology [65, 66].

Further techniques have been subsequently introduced, which helped cancer studies in zebrafish to progress. The aim was to create loss-of-function phenotypes or to introduce transgenes that are typically mutated in human cancer into fish models. Research in this field brought forward evidence that many mutated tumor suppressor genes, such as Tp53, and oncogenes, such as mMyc and KRas, could generate parallel tumors in zebrafish in the same way as they had been observed in humans. This shed light on the evolutionary conservation of drivers and pathways of tumorigenesis between man and fish [67].

Currently, the most used techniques for gene manipulation in zebrafish are morpholino oligomers (MOs) [68], zinc finger nucleases (ZFNs) [69], transcription activator-like effector nucleases (TALENs) [70] and the CRISPR (clustered regularly interspaced short palindromic repeats) system [71]. However, new promising techniques have recently been introduced to be used in zebrafish, such as TEAZ (transgene electroporation in adult zebrafish) and tumor cell transplantation, especially in the form of PDX (patient-derived cancer cell xenotransplantation).

MOs are small synthetic oligomers that block mRNA translation in vivo; they are easy to use and enable us to obtain models in a short amount of time, despite concerns about their off-target effects and their inexact reproduction of genome-editing mutants, thus requiring a control with the knockout phenotype [68, 72, 73].

ZNFs is a useful technique for multiplex gene targeting to be performed in one round, either creating knock-outs (loss of function) or knock-ins (gain of function) [69].

TALENs enable us to produce heritable gene disruptions in the vertebrate genome; more importantly, they can create mutations in somatic tissues with a high success rate, including bi-allelic mutations [70].

CRISPR/Cas9 is a technique in which Cas9 endonuclease recognizes a specific DNA sequence by means of a guide RNA sequence binding both DNA and Cas9. Zebrafish models based on this technique are widely used today due to the potential possibility to target multiple genes at the same time and to its high efficiency [71, 74, 75].

TEAZ is a new technique that enables the injection of DNA constructs containing tissue-specific promoters and genes of interest into adult tissue. In addition, TEAZ is extremely fast as far as tumor onset is concerned, and the expression of genes of interest can be evaluated in adult fish [76]. TEAZ is very promising compared to conventional zebrafish cancer models created by means of the aforementioned techniques. In fact, the latter involves the injection of nucleic acids into one-cell stage embryos. Therefore, it is sometimes difficult to study cancer pathogenesis and development in animal models, since the onset and the site of the developing tumors are not accurate, and the spreading of metastases could be hard to evaluate. Alongside TEAZ, cancer cell transplantation in zebrafish embryos and adults could partially overcome the problems connected with common techniques [77].

Tumor cell transplantation is an important tool in studying tumor invasiveness. It involves cancer cell transplantation from a donor to a recipient of the same species (allograft) or of a different species (xenograft) [78].

Many studies have demonstrated that zebrafish embryos can engraft human cancer cells and give precious insight into disease pathogenesis.

As for human cancer xenotransplantation, zebrafish have some advantages compared to murine models, especially because a high number of transparent embryos lacking a mature immune system can be transplanted with cancer cells and tracked. In other words, visualization of cell-cell interactions in vivo is possible in zebrafish. Moreover, PDX in zebrafish can help us find new targets for targeted anti-cancer treatments. There is evidence that pre-clinical research might shorten the time for drug approval, mostly due to drug re-purposing [10]. The zebrafish has already shown to be a reliable model to assess drug efficacy and sensitivity, since in some experiments patient-derived cells responded well to the same drugs that were used in patients [79].

Thus, the use of zebrafish as a pre-clinical screening model for patient-derived cancer cell xenotransplantation might revolutionize our approach to cancer, especially in a personalized medicine perspective, and explains the growing interest in PDX studies in zebrafish [77].

The variety of cancer types that have been successfully reproduced in zebrafish prove that this animal model has a lot of potential in the analysis of almost every type of cancer observed in humans. Genetic models of cancer in zebrafish include peripheral nerve sheath tumor (PNST) [80–82], rhabdomyosarcoma (RMS) [83, 84], melanoma, [85–90] thyroid cancer [91], pancreatic cancer [92, 93], hepatocellular carcinoma (HCC) [94–96], intestinal tumors [97, 98], testicular tumors [99], T-cell acute lymphoid leukemia (T-ALL) [83, 100–102], Acute Lymphoid leukemia (AML) [103–106], chronic myeloid leukemia (CML) [102, 107], myelodysplastic syndrome (MDS) [108].

Some of these cancer types, along with others, have been studied with PDX in *Danio rerio* [10]. Interestingly, the zebrafish has proved to be a reliable model for PDX for some cancers that develop in human organs that fish do not have, such as the breast, prostate and lungs [109, 110]. It has proved to be a good model for studying rare cancer pathogenesis as well, such as Ewing sarcoma [111].

### Melanoma models in zebrafish

To better understand the mechanisms underlying melanoma, the zebrafish represents an excellent model through the use of xenografts [112] and transgenic models [113, 114].

Melanoma has certainly been one of the most studied cancers and the most analyzed skin cancer in zebrafish, since the first description of BRAF V600E model. It is known that the V600E mutation, a key melanoma driver found in about 43–50% of melanomas [10, 115, 116], is also frequently found in benign *naevi* and moles which do not progress to cancer. It is also known that the loss of function of the tumor suppressor gene p53 (p53−/−) is required for cancer progression in *naevi*. However, the long time lapse and rarity of melanoma tumor formation (one to three in a fish’ lifetime) in zebrafish carrying both BRAF V600E and p53−/− mutations, imply that there are other molecular alterations and pathways playing a role in melanoma formation. Based on the observation that crestin, the expression of which is generally limited to neural crest progenitor cells in developing zebrafish embryos, was expressed in zebrafish melanomas [117], studies were performed in which engineered transgenic zebrafish expressing GFP (green fluorescent protein) under the control of crestin-regulatory elements were tracked. GFP-positive cells showed that only individual melanocytes that reactivated crestin could initiate melanomas. This highlighted that melanoma at a one-cell-state is based on reprogramming the cell to become more neural-crest-like [86]. As confirmation, consistent with crestin expression was the expression of the SOX10 transcription factor, a conserved early neural crest marker that helps melanocyte reprogramming to an embryonic state. Reactivation of neural crest genes such as crestin (in zebrafish melanoma) and SOX10 (in zebrafish as well as in human melanoma cell lines) is probably consequent to epigenetic modifications on histones, as shown by some histone markers known as super-enhancers [117].

N-RAS mutation has also been studied in zebrafish and its expression led to hyperpigmentation throughout the zebrafish’s body. When p53 mutation was added to mutated N-RAS, the fish developed invasive melanomas which were histologically and genetically correlated to human melanomas.

Although less frequent in melanomas than the previously mentioned BRAF and N-RAS mutation, H-RAS-mutated zebrafish models also displayed melanoma development [90, 118, 119].

Combining these assets with the excellent melanoma models engineered in zebrafish has led to several significant advances in our knowledge of melanoma behavior and molecular asset. New frontiers involve testing even infrequently mutated potential drivers, thus broadening the available models of cutaneous melanoma and introducing non-cutaneous melanoma zebrafish models [120]. Moreover, loss of function CRISPR/Cas9 gene targeting technology has been successfully used to create loss of function models, allowing testing of candidates that may alter disease onset and/or progression.

As an example, this technique was used to investigate SPRED1 function as a tumor suppressor in the context of KIT mutations in mucosal melanoma. SPRED1 knockdown, determining MAPK activation, conferred resistance to drugs inhibiting KIT tyrosine kinase activity. MAPK inhibition in SPRED1-deficient melanomas could therefore be a therapeutic hint and again proves the power of zebrafish modeling to investigate genetic interactions in cancer pathways [121].

Concerning the aforementioned cancer intra-tumoral heterogeneity, single cell RNA sequencing (sc-RNA sq) technologies provide an insight into melanoma complexity [122]. Analysis of cell dynamics at the minimal residual disease (MRD) stage, when persistent cells in otherwise disease-free tissue acquire specific properties for melanoma progression, proves fundamental to grasp the tumor vulnerability at a crucial point [123]. Sc-RNA sq was used to study MITF-low state role in melanoma progression in zebrafish genetic models with low activity of Mitfa, proving that very low or absent MITF activity characterized a residual disease like therapy-resistant melanoma [124]. Additional research on melanoma cells interaction with their microenvironment has been accomplished in a transgenic zebrafish model, proving the power of tools such as spatially resolved transcriptomics, sc- RNA-seq, and single-nucleus RNA-seq [125].

Interaction with metabolism has rarely been considered as an impacting factor in cancer and, more specifically, in melanoma; however, interference with liver gluconeogenesis has been successfully investigated in a zebrafish melanoma model through isotope tracing, confirming versatility of zebrafish in the field of research [126].

Not only has the zebrafish model helped to investigate melanoma genesis and development as far as its genetics is concerned, but also it has recently offered an insight into new therapeutic strategies for melanoma metastatic progression by targeting specific signaling cascades. For instance, human epidermal growth factor receptor (EGFR) signaling was implied when PLD c GMP analog protein kinase G activator 5 (PA5) was injected into zebrafish melanoma models, thus targeting the cGMP/protein kinase G pathway [127]. Another receptor tyrosine kinase, Xrmk, was identified as closely related to EGFR, and therefore involved in melanoma development and progression; in detail, Xrmk has been studied in *Xiphophorus* platyfish and in zebrafish as a therapeutic target [128]. Moreover, the activation of CD271, a member of the tumor necrosis factor receptor (TNFR) family, using a short β-amyloid-derived peptide, combined with chemotherapy or MAPK inhibitors, proved to significantly reduce metastasis in a zebrafish xenograft model [129].

### Squamous cell carcinoma models in zebrafish

Even though non-melanoma skin cancer in fish is less common compared to melanoma, zebrafish have been adequately used as a model to study the underlying pathogenetic mechanisms in these kinds of cancer as well.

Recent works that employ the SCC xenograft model in zebrafish have identified key molecules involved in the pathogenesis of squamous cell carcinoma (SCC) [130], as well as compounds that may be used as targets for SCC therapy [131]. A crucial molecule to be studied as a therapeutic target is the tyrosine kinase receptor Axl, which is highly expressed in SCC [132]. Other important targets are the COL7A1 gene, which is responsible for the development of aggressive SCCs in epidermolysis bullosa, and the recombinant type VII collagen (hrCol7), which is able to reverse SCC angiogenesis in the zebrafish model [133].

Another interesting in vivo xenograft model study has analyzed the role of the tyrosine kinase discoidin domain receptor 2 (DDR2) in cell proliferation, adhesion, differentiation and invasion in head and neck squamous cell carcinoma (HNSCC) [134]. The study shows that dasatinib, a Food and Drug Administration (FDA)-approved inhibitor of c-Kit, Proto-oncogene tyrosine-protein kinase (ABL, SRC) and Abelson murine leukemia viral oncogene homolog, may be potentially used in DDR2-positive SCC patients to block tumor cell invasion and migration [134].

Another potential compound for HNSCC treatment is the marine microbial extract luminacin. Studies in zebrafish embryos have shown that luminacin treatment of tumor cells stimulates autophagy in SCC cell lines, thus inhibiting cancer growth and progression [130].

Lastly, the zebrafish model has also been used to show that Flotillin-1 over-expression in KB cells (a subline of the keratin-forming tumor cell line HeLa) boosts KB cell motility and cell growth [135].

These studies prove that the zebrafish model may be adequately used not only in the evaluation of molecular pathways involved in SCC development and progression, but also in drug toxicity and screening assays.

### Other dermatological applications of zebrafish

Zebrafish can be used to study not only cancer derived from melanocytes, but also other disorders of melanogenesis, since melanogenesis pathways are conserved between zebrafish and mammals, and melanogenesis is a visible process in zebrafish embryos and in transparent *casper* adults [136]. Studying zebrafish albinism models, researchers could clarify the function of genes whose role in the pathogenesis of this disorder remains concealed and that might not yet be recognized as implicated in human albinism. Correct genetic diagnosis might prove crucial in treatment of different, but often clinically indistinguishable, forms of albinism. Thus, rapid CRISPR screening for gene function makes zebrafish an excellent model for albinism gene discovery. Though counterintuitive, zebrafish albinism models could also help clarify chemotherapeutic resistance mechanisms in cancerous melanocytes in melanoma [137, 138].

Hereditary pigment disorders have been investigated using MOs to ascertain the function of specific genes that had previously been identified in affected individuals. Protein O-fucosyltransferase 1 (pofut1) and presenilin enhancer-2 (psenen) knockdown zebrafish both displayed abnormal distribution in pigmentation, thus confirming involvement of the aforementioned genes in certain clinical presentations of Dowling-Degos syndrome, also known as reticulate pigmented anomaly of flexures. Furthermore, *oca2*-mutant zebrafish and c10orf11 knockout zebrafish were created to explore oculocutaneous albinism-related gene function in vivo, confirming involved conserved gene function throughout fish, mouse and humans. Hypopigmentation characterized also *snow white* zebrafish mutant carrying a *hps5* gene mutation, reproducing Hermansky-Pudlak syndrome (HPS) in fish models. Zebrafish *fade out* mutant also recreated HPS phenotype indicating that *fade out* gene could have a role in the pathogenesis of HPS. Disorders of copper metabolism were also reproduced in zebrafish with the *calamity* and *catastrophe* mutant models, underlying the influence that copper and, potentially, other nutrients, could have on melanin synthesis in melanocytes. Impact of stress on vitiligo development in fish was reproduced by treating zebrafish with interleukin-17, which determined altered pigmentation and autophagy in pigment cells [138].

Mutation in NRAS resulting in an I24N amino acid substitution was identified in an individual bearing typical Noonan syndrome features. N-Ras-I24N expressing zebrafish displayed developmental defects which were parallel to other Noonan syndrome-associated genes in zebrafish. Activation in N-RAS signaling pathway was therefore confirmed to be associated to a Noonan Syndrome phenotype. Of note, MEK inhibition completely rescued the activated N-Ras-induced phenotypes, confirming the exclusive mediation of Ras-MAPK signaling in the genesis of the syndrome [139].

Co-occurrence of Mongolian blue spots with vascular birthmarks defines a group of syndromes known as phakomatosis pigmentovascularis. Association with activating mutations in GNA11 and GNAQ genes, encoding a Ga subunit of heterotrimeric G proteins, was discovered and confirmed in a transgenic mosaic zebrafish model expressing mutant GNA11R183C under *mitfa* promoter, which developed extensive dermal melanocytosis recapitulating the human phenotype. Specifically, zebrafish embryos were injected with wild type human GNA11, GNA11R183C, or GNA11Q209L expressed under control of the melanocyte *mitfa* promoter. The embryos were grown to adult fish; their status of genetically mosaic animals was clinically visible as melanocyte patches, which received histological confirmation [140].

RASopathies result from germline mutations of the Ras/MAPK pathway. Systematic predictions on disease progression are not yet possible, even though available technologies in genome sequencing allow to identify multiple disease-related mutations. Nevertheless, zebrafish embryos represent a valuable model in assessing mutational effects. Jindal et al. succeeded in ranking several MEK1 mutations, proving that those found in cancer were more severe than those found in shared by RASopaties and cancer. Also, the latter resulted as more severe than those characterizing only RASopaties. A conserved ranking was observed in Drosophilaand the ranking could predict the drug dose to correct the defects [141]. Wound healing and re-epithelialization of adult zebrafish skin have been analyzed in several studies. In zebrafish the process of wound healing results in minimal scar formation. The process comprises a series of events: rapid re-epithelialization; migration of inflammatory cells; formation of granulation tissue consisting of macrophages, fibroblasts, blood vessels, and collagen; granulation tissue regression. Major steps and principles of cutaneous wound healing seem to be the same in adult mammals and adult zebrafish, thus making the zebrafish a valuable model for studying vertebrate skin repair [142]. Richardson et al. studied the wound healing process by creating full-thickness wounds with a laser on the flank of adult zebrafish in a rapid and reproducible way, confirming that the zebrafish is a unique and cost-effective model for skin repair [142]. Absence of wound scars in zebrafish, as observed in human embryos, due to the lack of the blood-clotting phase and to specific signaling mechanism, represents an attractive model to study healing processes and is expected to help to formulate an appropriate drug for cutaneous wound healing [143].

The aforementioned similarities of the zebrafish integument structure together with those of the inflammation mechanisms, make this teleost a fundamental and cost-effective model also to study major dermatologic inflammatory diseases, such as psoriasis. Several models, including mutant, morphant and environmentally inducible models, were created to investigate genetic alterations and molecular mechanisms of psoriasis [144–152].

## Conclusions

Inflammatory and neoplastic skin disorders are very common and are increasing worldwide.

**Fig. 2:**
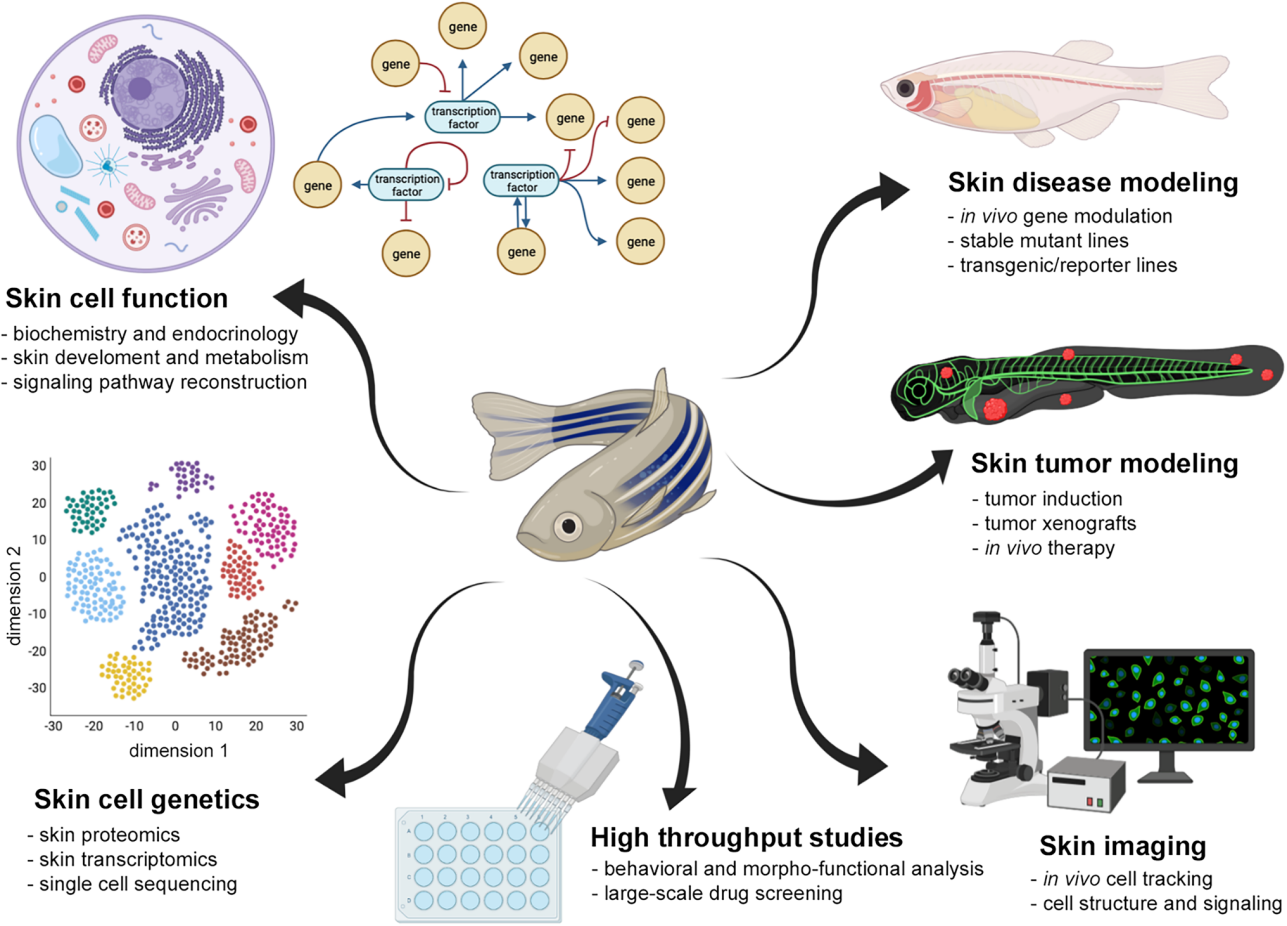
Zebrafish applications in skin biology. Examples of applications of the zebrafish model in the field of skin biology include skin disease and tumor modeling, biochemical and genetic tests, drug screen and in vivo imaging, all suitable for large-scale studies. Images created with BioRender.com.

Zebrafish can provide a suitable animal model to extend our understanding of the molecular and cellular mechanisms of skin disorders and to develop new therapeutic strategies in dermatology (Fig. 2). Zebrafish models of major interest in dermatological research are summarized in Table 1 [22, 39, 85–87, 89, 90, 147, 153–167].

**Table 1:** Main zebrafish models of dermatological interest

Tumor type/cutaneous condition	Involved gene/pathway or study drug	Technique	References
Oral squamous cell carcinoma	Sandensolide at different concentrations	Human cancer xenograft in 48 hpf embryos; incubation with different sandensolide concentrations	Yu et al. 153]
Epidermal adhesion mechanism deficit (Kindler syndrome, KS)	Kindlin-1 (regulator of integrin function)	Morpholino oligomers (MOs); stably mutated fish line	Postel et al. 154]
Epidermal stratification; keratinocyte proliferation and differentiation	TAp63 and p53	Target-selected mutagenesis (TILLING) and TEAZ (transgene electroporation in adult zebrafish)	Fischer et al. 22]
Psoriasis	Homozygous m14 mutant	EMS-induced mutagenesis; whole-mount immunohistochemistry	Webb et al. 147]
Cytokine function	IL-1β, TNF-α IL-6, IL-2, IFN-γ, IL-4/13, IL-10, IL-17 A/F, IL-21, IL-22, TGF-β1, IFN and TNF-α	Knock-down with morpholinos and overexpression of G-CSF by injection of eggs with in vitro transcribed cytokines mRNA	Zou et al. 39]
Melanoma	Nodal signaling	Human cancer xenograft transplantation models in zebrafish blastula 3hpf	Topczewska et al. 155]
Melanoma	*BRAFV600E tp53M214K*	Genetic model (BRAF/p53)	Patton et al. 85]
Melanoma	*BRAFV600E tp53M214K; crestin:EGFP*	Genetic model (BRAF/p53, crestin gene-reactivation). Crestin-based reporter transgene	Kaufman et al. 86]
Melanoma	*BRAFV600E mitfavc7*	Genetic model (BRAF plus conditional MITF mutation)	Lister et al. 87]
Melanoma	Oncogenic HRAS under the kita promoter; RAS-induced microRNAs (miR-146a and 193a) targeting Jmjd6	Genetic model (injection of fertilized eggs with plasmids)	Santoriello et al. 90] Anelli et al. 89]
Melanoma	*SETDB1* *BRAF, tp53*	Eϖpigenetic regulators in zebrafish cancer; miniCoopR vector system	Ceol et al. 156]
Melanoma	*BRAFV600E*, tp53−/−	Cancer allograft transplantation model in rag2E450fs, jak3P369fs, and prkdcD3612fs mutant fish. Immunocompromised transparent (*casper*) adult fish (primary cells)	Tang et al. 157, 158] Moore et al. 159]
Melanoma	*BRAFV600E*, tp53−/−	CRISPR technologies to modify zebrafish melanoma cell lines and the *casper* recipient. Cancer allograft transplantation model in transparent (*casper*) adult fish and in 48-hpf embryos	Heilmann et al. [160]
Melanoma	*BRAFV600E*, tp53−/−	Cancer allograft transplantation model in transparent (*casper*) fish	Benjamin et al. [161]
Melanoma	*BRAFV600E*, tp53−/−	Tracking of zebrafish melanoma extracellular vesicles	Hyenne et al. [162]
Melanoma	*BRAF, KRAS*	Human cancer xenograft transplantation models in zebrafish blastula	Lee et al. [163]
Melanoma	*BRAF, PTEN*	Human cancer xenograft transplantation in 2-dpf embryos	Haldi et al. [164]
Melanoma	BRAF, p53, ras-MAPK pathways	Murine cancer xenograft transplantation models in 48-hpf embryos	Nicoli et al. 165] Nicoli et al. [166]
Melanoma	*BRAFV600E*	Human patient-derived cancer xenograft transplantation (PDX) models in two-month-old immunocompromised zebrafish	Yan et al. 167]

Owing to its low maintenance cost, highly conserved genome, and easy genetic manipulation, the zebrafish is an excellent model for preclinical research in dermatological laboratories, thus bridging the gap between in vitro cell culture an in vivo mammalian models.

## Methodological approach

The database of Pubmed was queried with the following search string (zebrafish OR/AND dermatology* OR skin cancer* OR melanoma*) under all fields (last search December 2021).
